# Red Blood-Globules

**Published:** 1867-07

**Authors:** G. W. Decamp

**Affiliations:** Mansfield, O.


### DENTAL REGISTER.

Vol. XXI.] JULY, 1867. [No. 7.

### Original Communications.

### RED BLOOD-GLOBULES.

BY G. W. DECAMP, D.D.S., MANSFIELD, O.

Read before the Central Ohio Dental Association.

Gentlemen:—I do not expect to present any unknown truth, nor to do more than simply call your attention to a subject which is, to a certain extent, clouded with mystery.

A thorough knowledge of the constituents of the blood is of importance to an intelligent Dentist. It is clearly seen that the condition of the blood exerts an influence on the teeth, as well as on other parts of the body; and all will agree that we should not only know what articles of food are best for it, but that we should also know its composition and formation.

The blood is a non-homogeneous fluid, and is easily distinguished from the other fluids of the body by its color. Its taste is slightly saline, its reaction slightly alkaline, its odor peculiar, and it is somewhat unctuous to the touch. Its specific gravity varies, being usually about 1.055. It is composed of red and colorless corpuscles, fibrin, albumen, fatty matter, salts, mineral substances and water. In bulk, the corpuscles exist in the proportion of about 512 parts of moist corpuscles to 488 parts of the liquor sanguinis in a thousand. A slight variation either above or below this proportion of moist globules is not generally incompatible with a good state of health.

According to Lehman and others, the average weights of the constituents of the blood, in a thousand parts, are, corpuscles, 149½ parts; fibrin, 2 parts; albumen, 39½ parts; fatty matter, 2 parts; salts and mineral substances, 8½ parts; extractive matter, 3 parts; water, 795½ parts.

The red globules vary in size, their transverse diameter being from $\frac{1}{3000}$ to $\frac{1}{4000}$ of an inch. In form they are spheroidal, with a depression on either side. They are from $\frac{1}{14000}$ to $\frac{1}{15000}$ of an inch in thickness, or about $\frac{1}{5}$ of their transverse diameter.

As to the structure of red blood-globules, there has been and is a wide difference of opinion. Dalton considers them homogeneous in structure, consisting of a mass of organized animal substance, he also says: "They have sometimes been erroneously described as consisting of a closed vesicle or cell-wall containing in its cavity some fluid or semi fluid substance of a different character from that composing the walls of the vesicle itself. No such structure, however, is to be seen in them."

Again, he says: "They are of the same color, consistency and composition throughout."

Other prominent writers differ with Dalton. Williams believes the red corpuscles of the blood to be formed of delicate hollow films of albuminous substance, containing, in their interior cavity, a liquid compound of two peculiar azotized principles mingled together. Thus, we see the opinion of Dalton—"That in color, consistency and composition, the red blood-globules are the same throughout"—is in direct opposition to the views of other medical authors. Which is right I leave for you to judge.

The blood globules, according to Lehman, are found to be composed in a thousand parts: water, 688 parts; globulin,

282.22 parts; hæmatine, 16.75 parts; fatty substances, 2.31 parts; extractive matter, 2.60 parts; chloride of sodium, chloride of potassium, phosphate of soda, phosphate of potassa, Sulphate of soda, sulphate of potassa, phosphate of lime and magnesia, 2.12 parts.

Globuline and Hæmatine are the more important ingredients. Globuline is about 16¾ times more abundant than Hæmatine, it is an albuminous fluid, is soluble in water, coagulates at a temperature from 158° to 200° F.

Hæmatine is the coloring matter of the blood globules. It is composed of carbon, oxygen, nitrogen, hydrogen and iron, the latter of which composes from six to seven per cent. It is generally considered to be the substance which nourishes the muscular tissues and nervous filaments of the body. The red blood-globules contain the greater part of the phosphate salts, phosphorous and fat, which are largely used in the formation of the nervous and muscular system.

The proportion of red globules found ordinarily in the human blood varies. Williams gives from 110 to 152 in a thousand. Red globules are found to be more abundant in early adult life than at any other period. The proportion is generally from one to two per cent. less in healthy females than in healthy males.

As to the origin of the red globules, little is known, and after carefully searching our best authors upon this subject, we are unable to draw a definite conclusion. It is believed by some that the red globules are matured colorless corpuscles. Dr. Beal's views upon this subject, to be found in the Edinburgh Medical Journal and Journal of Microscopic Science, Vol. 4, 1864, are as follows: Applying his general views upon "germinal matter" and "formed material" to the blood, believes that the white blood-corpuscle consists of the former and red blood-corpuscle of the latter, and that in red corpuscles containing nuclei, the nucleus represents the germinal matter. Dr. Beal does not believe in the existence of cell-walls. He takes, for illustration, the red blood-corpuscles of the frog, the outer part, which can not be colored by carmine, is "formed material" which was once "germinal matter;" he also believes that "germinal matter" is capable of producing matter like itself, and of being resolved into colored "formed material," but that "formed material" is not capable of forming material like itself. It is the seat of chemical and physical action alone. Vital changes are restricted to the "germinal matter."

Dr. Beal states that when the blood becomes stationary, the white corpuscles and nuclei of the red increase in size and subdivide. In inflammation of the vesicles of the frog's foot, the increase of white corpuscles is not alone dependent on the arrest of the circulation, but the corpuscles actually increase in the clot.

Dr. Williams, in reference to the formation of the red globules, says: "During early embryo life they unquestionably increase by a process of subdivision. Each then contain a nucleus cell which parts into two, a new corpuscular vesicle being then developed round either half. In more mature life this multiplication by division does not appear to be continued. There is no nucleus whatever in the complete adult red corpuscle of the human blood. The red corpuscles seem, then, to be formed out of the chyle and lymph globules through some unknown course of transmutation."

In regard to the supposition that the colorless corpuscles change into the colored, he believes there to be a greater probability in the notion that the colorless and colored corpuscles are distinct and independent formations, designed for distinct offices, but both having a common origin—the chyle and lymph globules. Their development, growth and decay depend greatly upon the condition of the organs of nutrition, and also on the lungs, liver, and other glands, also on a proper supply of the ferruginous element of which they are largely composed—(Dr. Mann.)

Dalton, as to the formation of the red globules, differs in part from the opinions of the authors given. He considers the red and colorless globules as distinct and independent elements. "There is no probability that the red globules are produced or destroyed in any particular part of the body. One cause for the belief that the red corpuscles were produced by a metamorphasis of the colorless globules, was the supposition that the red globules were continually destroyed in some part of the circulation, and, therefore, they must be reproduced to counterbalance their destruction. But there is no reason for believing that the real globules, as anatomical forms would be any less permanent than the nervous filament, or the muscular fiber. They undergo a constant change, it is true, like all the constituent parts of the body, an interstitial metamorphosis. They absorb nutritious substances from the blood, and give to the circulating fluid other substances which result from their internal waste and disintegration. Anatomical forms never undergo destruction or renovation, but only the proximate principles of which they are composed. The effect of this interstitial nutrition in the blood globules is merely to keep them in a healthy condition. Their component parts are continually changed by decomposition and disintegration, as they pass through the body, but the globules retain their form and texture and still remain a constituent part of the blood.

Thus we see, in the theories given, a great difference of opinion among our best authors as to the structure, composition and origin of the red blood-globules; and after a careful search for definite information, we are forced to the conclusion that little is written which gives us a perfect knowledge of this important constituent of the blood.

Would it not be profitable for us, as members of this branch of medicine, to investigate this subject farther, that we may, if possible, be more useful, and also take our rank with other parts of our profession.

